# Late‐Onset Suspected Coronary Vasospasm Following Focal Monopolar Pulsed‐Field Ablation for Atrial Fibrillation

**DOI:** 10.1111/jce.70370

**Published:** 2026-05-15

**Authors:** Yui Araki, Kanae Hasegawa, Ryohei Nomura, Hiroshi Tada

**Affiliations:** ^1^ Department of Cardiovascular Medicine, Faculty of Medical Sciences University of Fukui Fukui Japan

## Abstract

**Introduction:**

Coronary vasospasm is a recognized complication of pulsed‐field ablation (PFA), but delayed events are rarely described.

**Methods and Results:**

A 64‐year‐old man underwent atrial fibrillation ablation using a focal monopolar PFA catheter. He developed transient chest discomfort 18 h after ablation, followed by sustained chest tightness with ST‐segment depression 42 h post‐procedure. Symptoms and electrocardiographic changes resolved promptly with sublingual nitroglycerin, suggesting coronary vasospasm.

**Conclusion:**

Coronary vasospasm after focal monopolar PFA may occur beyond the immediate post‐procedural period, which may have implications for monitoring and discharge planning.

## 1 Case Presentation

A 64‐year‐old man with atrial fibrillation underwent catheter ablation. He was receiving amlodipine (10 mg daily) for hypertension. He had no prior history of angina, including vasospastic angina. Transthoracic echocardiography demonstrated no structural heart disease, including no left ventricular hypertrophy. Preprocedural contrast‐enhanced computed tomography revealed no abnormalities in the coronary arteries and a common trunk of the left pulmonary veins.

The procedure was performed under moderate sedation and analgesia with thiamylal, dexmedetomidine, and fentanyl. Pulmonary vein isolation (PVI) was performed sequentially, beginning with the right pulmonary veins, followed by the left pulmonary veins, using a Sphere‐9 catheter with the Affera mapping system (Medtronic, Minneapolis, MN, USA). Complete isolation of the right pulmonary veins required 32 pulsed‐field ablation (PFA) applications, whereas isolation of the left pulmonary veins required 25 PFA applications. Therefore, additional posterior wall isolation with roof and bottom lines was performed (14 and 16 applications to the roof and bottom lines, respectively). After completion of ablation (Figure 1A,B), demonstrating left atrial voltage mapping and ablation tags), left atrial mapping demonstrated complete PVI with no residual pulmonary vein potentials. Electrical isolation of the posterior wall was further confirmed by the absence of left atrial capture during high‐output pacing from the posterior wall. High‐rate pacing from the coronary sinus under isoproterenol infusion failed to induce any atrial arrhythmias, and the procedure was concluded. No complications were observed during the procedure, and the patient was returned to the ward.

**Figure 1:**
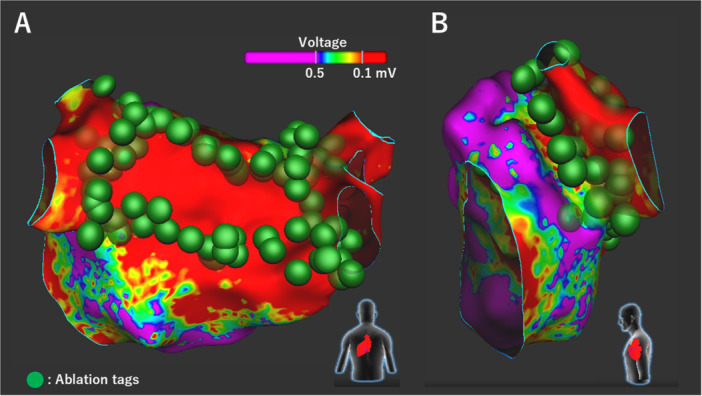
Left atrial voltage map demonstrating with ablation tags showing pulsed‐field applications for pulmonary vein isolation and additional posterior wall isolation, including roof and bottom lines. (A) Posterior‐anterior view of the left atrium. (B) Left‐right view of the left atrium.

At 7:00 on postoperative day (POD) 1, 18 h after the procedure, the patient experienced transient chest discomfort lasting approximately 10 s; however, no electrocardiographic changes were observed. Subsequently, at 7:36 on POD 2 (24 h later), the patient developed intermittent chest pain, lasting approximately 5 min, that progressed to continuous distress, accompanied by ST‐segment depression on a 12‐lead electrocardiogram (Figure 2A). Sublingual nitroglycerin (one tablet) promptly relieved the symptoms and resolved the ST‐segment depression completely (Figure 2B). The patient was discharged on POD 2 in the afternoon. At discharge, the patient declined additional coronary vasodilators; therefore, only sublingual nitroglycerin was prescribed on an as‐needed basis. Amlodipine was continued for hypertension. Post‐discharge, the patient experienced resting chest pain (triggered by stress or tension) identical to symptoms on POD 2. Initially occurring every 3 days, these episodes gradually diminished in frequency and intensity, resolving completely 2 months after the procedure.

**Figure 2:**
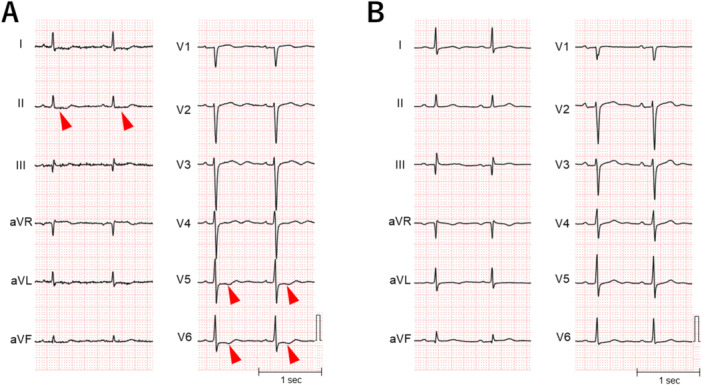
Electrocardiographic changes during vasospastic angina. (A) Twelve‐lead electrocardiogram showing ST‐segment depression during sustained chest pain approximately 42 h after ablation, higlighted by red arrowheads, with blood pressure at 130/80 mmHg. (B) Complete resolution of ST‐segment depression after sublingual nitroglycerin administration, with blood pressure at 120/78 mmHg.

## 2 Discussions

This case report details a suspected occurrence of coronary vasospasm 42 h following atrial fibrillation ablation with a focal monopolar PFA catheter. While angiographic evidence of vasospasm was not obtained, the clinical symptoms‐such as transient chest pain, ischemic ST‐segment changes, and swift relief with nitroglycerin‐strongly support a vasospastic origin.

While PFA is regarded as tissue‐selective and less prone to inducing permanent collateral damage, coronary vasospasm remains a clinically significant concern. Previous studies have documented instances of coronary spasm occurring during or shortly after PFA, predominantly with single‐shot systems [1]. Regarding focal PFA catheters, only two cases have been reported [2, 3], both of which manifested acutely during the procedure. In contrast, our patient exhibited a brief prodromal symptom 18 h post‐procedure, followed by a prolonged symptomatic episode with ischemic ST‐segment depression approximately 42 h after ablation. These observations indicate that PFA‐related coronary vasospasm events may extend beyond the immediate post‐procedural period.

Recent evidence further supports this concept. Gunawardene et al. [4] reported life‐threatening delayed myocardial ischemia and malignant arrhythmias, including sudden cardiac death occurring up to 22 days after PFA. Notably, most cases involved ablation beyond PVI, including posterior wall isolation [4]. In addition, large registry data such as the MANIFEST‐PF study have identified coronary artery spasm as a potential, albeit rare, complication of PFA [5].

In the present case, posterior wall isolation was also performed in addition to PVI. Although the ablation sites were remote from the coronary arteries, PFA‐induced autonomic imbalance, endothelial dysfunction, or post‐ablation inflammatory responses may have contributed to delayed coronary vasospasm.

Notably, this patient was already receiving a calcium channel blocker, suggesting that vasospasm can occur despite baseline vasodilator therapy.

These findings support the concept that coronary vasospasm events after PFA may extend beyond the early post‐procedural period. In particular, even transient chest discomfort within the first 24 h may represent a prodromal sign of subsequent clinically significant vasospasm. These observations may have implications for post‐procedural monitoring strategies following PFA.

This case has several limitations. Coronary angiography was not performed during the episode, and therefore vasospasm could not be definitively confirmed.

## Funding

The authors have nothing to report.

## Disclosure

Hiroshi Tada: honoraria for lectures or speaker bureaus from DAIICHI SANKYO COMPANY Ltd., Medtronic Japan Co. Ltd., BIOTRONIK Japan Inc., and Boston Scientific Japan K.K. grants (Investigator initiated study unrelated to manuscript topic) from Abbott Medical Japan LLC.

## Conflicts of Interest

The authors declare no conflicts of interest.

## Data Availability

The data that support the findings of this study are available from the corresponding author upon reasonable request.
